# OR10-001 - Altered mitochondrial ROS and metabolism in TRAPS

**DOI:** 10.1186/1546-0096-11-S1-A185

**Published:** 2013-11-08

**Authors:** R Siegel, L Billingham, M pelletier, C Cudrici, A Ombrello, M Murphy, D Kastner

**Affiliations:** 1 NIAMS, Bethesda, USA; 2 NHGRI, NIH, Bethesda, USA; 3 Cambridge University, Cambridge, UK

## Introduction

Mutations in TNFR1 cause the familial autosomal-dominant autoinflammatory disorder TNF receptor-associated periodic syndrome (TRAPS). Most TRAPS-associated mutations in TNFR1 disrupt normal receptor function and cause retention of the mutant protein in the ER. Cells expressing mutant TNFR1 have enhanced MAP Kinase activation at baseline and hyper-responsiveness to innate immune stimuli, which is aided by the wild-type receptor. We have previously found that reactive oxygen species (ROS) generated by mitochondrial respiration is critical for this phenotype [1]. TRAPS patient cells, and cells from TNFR1 mutant mice display enhanced basal and maximal oxygen consumption, suggesting that enhanced mitochondrial respiration can in some circumstances contribute to acute inflammatory responses through increased generation of ROS.

## Objectives

To identify the metabolic and mitochondrial abnormalities underlying increased ROS production and hyper-inflammatory responses of TRAPS cells and more generally, the role of mitochondrial ROS in acute inflammatory responses and production of pro-inflammatory cytokines.

## Methods

Oxygen consumption, reactive oxygen species generation, and cytokine production was measured in mouse embryonic fibroblasts and macrophages from mice harboring TRAPS-associated TNFR1 mutations and peripheral blood monocytes from TRAPS patients. Agents that disrupt specific complexes in the electron transport chain (ETC) or import of key substrates into mitochondria were used to dissect the sources of increased ROS production, mitochondrial oxygen consumption and transcription of pro-inflammatory cytokines and chemokines after treatment with innate immune activating stimuli such as LPS.

## Results

Experiments with permeablized cells fed with ETC substrates showed no intrinsic abnormalities in complexes I-IV of the ETC. Fatty acid supplementation and etomoxir, which blocks fatty acid uptake by mitochondria, showed that fatty acid oxidation and delivery to mitochondria is a key driver of increased spare respiratory capacity in TNFR1 mutant cells. However inflammatory cytokine production in response to LPS is not reduced in the presence of etomoxir in normal cells or those harboring TNFR1 mutations. Rather, inhibition of glycolysis was more effective in reducing macrophage production of IL-6 and IL-1.

## Conclusion

Enhanced inflammatory cytokine production in TRAPS is a result of altered cellular metabolism rather than intrinsic abnormalities in the mitochondrial ETC. Increased mitochondrial fatty acid metabolism accounts for the increased mitochondrial respiratory reserve, but not inflammatory cytokine production in TNFR1 mutant or normal macrophages. These results emphasize the pathogenic role of mitochondrial ROS in inflammatory responses, which is is being investigated using mitochondria-targeted antioxidants in mouse models and a planned human trial of the mitochondrial antioxidant Mito-Q.

## Competing interests

None declared.
